# Preparation, Characterization and Application of Nano-Graphene-Based Energetic Materials

**DOI:** 10.3390/nano11092374

**Published:** 2021-09-13

**Authors:** Xiaolong Fu, Yonghu Zhu, Jizhen Li, Liping Jiang, Xitong Zhao, Xuezhong Fan

**Affiliations:** 1 Xi’an Modern Chemistry Research Institute, Xi’an 710065, China; fuxiaolong204@163.com (X.F.); jiangliping204@163.com (L.J.); zhao_xt204@163.com (X.Z.); xuezhongfan@126.com (X.F.); 2 Schoool of Chemistry and Chemical Engineering, Nanjing University of Science and Technology, Nanjing 210094, China; zhuyonghu@njust.edu.cn; 3 Institute of Space Propulsion, Nanjing University of Science and Technology, Nanjing 210094, China

**Keywords:** applied chemistry, energetic materials, nano-graphene, preparation, desensitization, combustion

## Abstract

Nano-graphene-based energetic materials, as a new type of composite energetic materials such as desensitizer and combustion catalyst, have attracted extensive attention from energetic researchers. In this paper, the preparation of nano-graphene-based energetic materials, the desensitization effect of nano-graphene-based on energetic compounds, the thermal decomposition and combustion behavior of the materials are reviewed. Meanwhile, the existing problems and future development of nano-graphene-based energetic compounds are discussed.

## 1. Introduction

In modern war, the increasingly harsh battlefield environment brings unprecedented challenges to the battlefield survivability of combatants and weapon platforms. It is urgent to improve the storage and use of the safety of weapons and ammunition, as well as the ability of anti-attack and anti-explosion, which makes insensitive high-energy materials become one of the research hotspots in the field of energetic materials [1,2]. The traditional high-energy propellants have high sensitivity and weak anti-stimulation ability, so they are easy to ignite accidentally when they encounter unexpected stimulation, and often become the prime factor of ammunition safety accidents.

In recent years, nano-graphene-based energetic materials, as a new type of composite energetic materials such as desensitizer [3,4,5] and combustion catalyst [6,7,8,9], have attracted extensive attention from energetic researchers. In terms of microstructure, graphene is a single or multilayer two-dimensional material composed of six-membered rings of carbon atoms, and this structure gives it extremely strong compressive resistance [10]. Due to the unique conjugated π bond structure in graphene, the electron motion in graphene is very unique, the carrier mobility of graphene is 10 times that of silicon [11]. Therefore, graphene has excellent properties such as high strength [12], electrical conductivity [13], thermal conductivity [14] and specific surface area [15], which have attracted great attention in the fields of propellants and explosives [16,17]. The special properties of graphene make it an excellent additive in significantly increasing the mechanical, thermal and electrical properties of composites materials [18,19].

In this paper, in order to provide a reference for the application research of nano-graphene-based energetic compounds, the preparation, desensitization, thermal decomposition and combustion properties of nano-graphene-based energetic compounds were reviewed.

## 2. Preparation of Nano-Graphene-Based Energetic Materials

The preparation of nano-graphene-based energetic compounds is the basis of the research of this compound. Researchers have carried out a lot of research on the preparation of nano-graphene-based energetic compounds.

At present, there are various graphene-based materials, and different uses have different requirements for graphene. The typical materials mainly include graphene-based ammonium nitrate compound, graphene-based metallic compound [20], graphene-based high nitrogen compound, et al.

### 2.1. Graphene-Based Ammonium Perchlorate Compound

**Figure 1:**
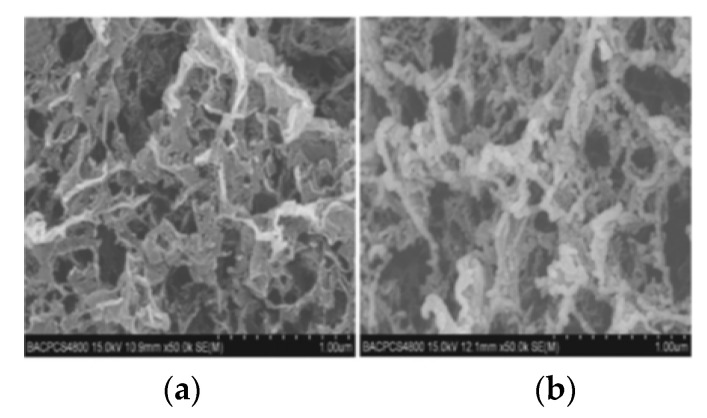
SEM images of graphene (**a**) and AP/graphene nanocomposites (**b**). Reprinted from ref. [21].

As the main component of high-energy composite propellant, Ammonium perchlorate (AP) also needs to be further improved in order to make it safer. Wang [21] et al. prepared an ammonium perchlorate/graphene nanocomposite based on oxidant ammonium perchlorate and graphene aerogel by sol–gel method. The ammonium perchlorate/graphene nanocomposite is similar in structure to graphene, but there are still many gaps inside it. The specific surface area was up to 49.2 m^2^/g, and a large number of ammonium perchlorate particles (average particle size 69.4 nm) were attached to the graphene skeleton. Figure 1 shows the SEM image of graphene and AP/graphene nanocomposites. From (a), it can be seen that GA has a rich pore structure, and from (b), it can be seen that many AP particles are adsorbed on the graphene framework. The results of the elemental analysis show that the content of ammonium perchlorate in the material can reach 94%, indicating that ammonium perchlorate/graphene is a very promising composite material as the main energy component of the propellant.

Lan et al. [22] prepared a novel graphene/Fe_2_O_3_/ammonium perchlorate nanostructured energetic material by sol–gel method and supercritical carbon dioxide drying technology based on ammonium perchlorate/graphene nanocomposites. The experimental results showed that Fe_2_O_3_ and ammonium perchlorate were evenly dispersed in the nano-graphene aerogel layer. The porosity (SSA) of graphene and graphene/Fe_2_O_3_/ammonium perchlorate nanocomposites are 717 m^2^ g^−1^ and 123 m^2^ g^−1^, respectively. The total pore volumes (Vtot) of graphene and graphene/Fe_2_O_3_/ammonium perchlorate were 3.37 cm^2^ g^−1^ and 0.46 cm^2^ g^−1^, respectively.

### 2.2. Graphene-Based CL-20 (Hexanitrohexaazaisowurtzitane) Compound

**Figure 2:**
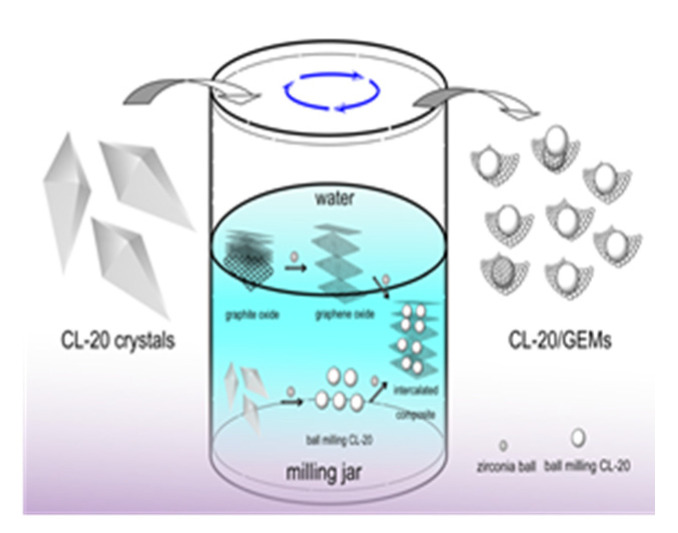
Schematic diagram of the formation of CL-20/graphene composite material. Reprinted from ref. [23].

Ye et al. [23] developed CL-20/graphene nanocomposites with the ball milling method, as shown in Figure 2 and they proposed that the formation of CL-20/graphene composites during ball milling could be divided into two processes: the peeling of graphite material and the refinement of CL-20, and the formation of sandwich composites.

**Figure 3:**
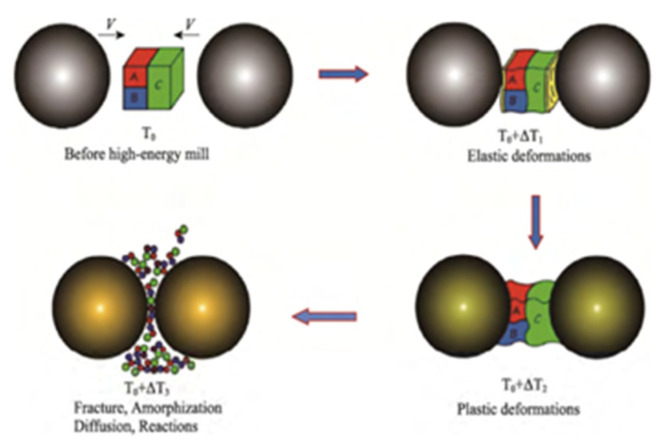
Schematic diagram of mechanochemical effects in the ball milling process. Reprinted from ref. [23].

The particle size of composites prepared by this method is generally about 7–8 microns. However, the energetic material may generate local hot spots during the ball milling process. Figure 3 shows the crushing and mixing process and the mechanochemical effects of the material during the ball milling process. At the same time, the energetic material may also undergo crystalline changes and other reactions under the influence of high temperature and high pressure, so the safety performance needs to be improved.

### 2.3. Graphene-Based HMX (Cyclotetramethylene Tetranitramine) Compound

**Figure 4:**
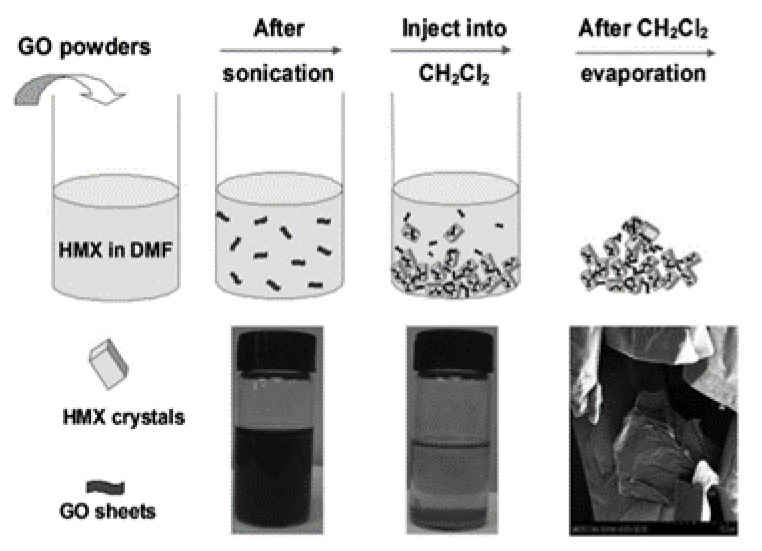
The production scheme of HMX/graphene composite material. Reprinted with permission from ref. [24]. Copyright 2013 John Wiley and Sons.

In a typical preparation process, HMX is dissolved in dimethyl sulfoxide at 40 °C and then graphene (about 2%) is added. Graphene was completely dispersed in a dimethyl sulfoxide solution of HMX by ultrasound, and CH_2_Cl_2_ (in which graphene is insoluble) was added to the mixture. After filtration, washing and drying at 40 °C, the HMX/graphene complex was obtained. The specific operation process is shown in Figure 4 [24]. It is found that the addition of graphene does not change the crystal shape of HMX, but the surface morphology of HMX and HMX/graphene is very different. The surface of the unmodified HMX crystal is smooth and clean, while some wrinkles are observed on the surface of the HMX/graphene complex, which can significantly improve the safety of HMX.

### 2.4. Graphene-Based Metallic Compound

**Figure 5:**
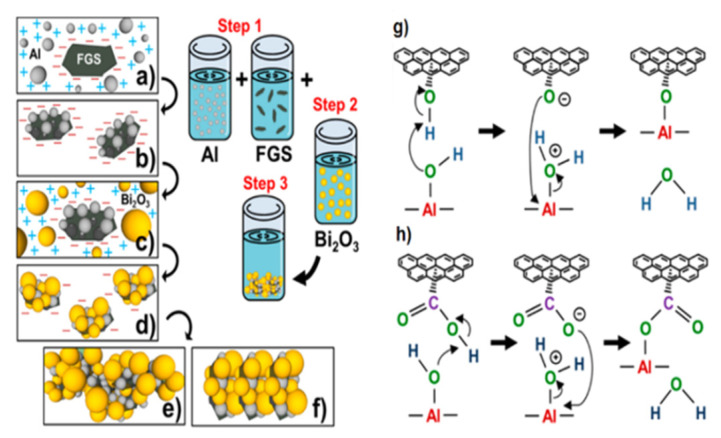
Schematic of the self-assembly process: (**a**) electrostatic attraction of Al to graphene, (**b**) covalent bonding of graphene/Al existing as a stable graphene/Al dispersion, (**c**) electrostatic attraction of Bi_2_O_3_ to graphene/Al nanostructures, (**d**) noncovalent assembly of Bi_2_O_3_ on graphene/Al; instability of graphene/Al/Bi_2_O_3_ dispersion continues the self-assembly process to form ultradense macrostructures, (**e**) The relative ratio of GO to Al and Bi_2_O_3_ drives the iterative self-assembly process of the macrostructures into layer by layer, (**f**) The relative ratio of GO to Al and Bi_2_O_3_ drives the iterative self-assembly process of the macrostructures into random orientations, (**g**) Chemical interactions between hydroxyl groups of GO and surface hydroxyl groups of Al nanoparticles leading to C–O–Al covalent bond, and (**h**) carboxylic groups of GO and hydroxyl groups of Al nanoparticles leading to O=C–O–Al covalent bond. Reprinted with permission from ref. [25]. Copyright 2014 American Chemical Society.

Thiruvengadathan et al. [25] independently developed the self-assembly method of Al and Bi_2_O_3_ particles on functionalized graphene sheets (FGS). The self-assembly process and the formation of chemical bonds are shown in Figure 5. The nanocomposite structure was formed in a colloidal suspension and eventually condensed into an ultra-dense macrostructure containing 5% of graphene.

### 2.5. Graphene-Based High Nitrogen Compound

**Figure 6:**
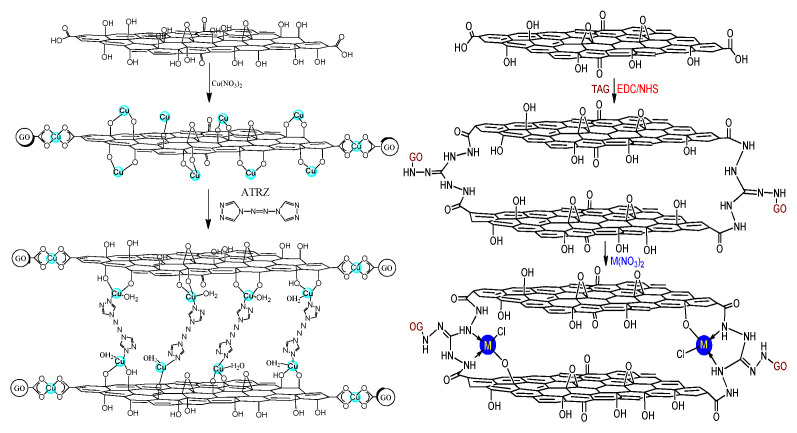
Graphene-Cu (II)-4,4′-azo 1,2,4-triazole and graphene-triaminoguanidine M were prepared by triaminoguanidine. Reprinted with permission from ref. [26]. Copyright 2016 American Chemical Society.

Gozin et al. [26] used 5,5′-azo-1,2,3,4-tetrazole (TEZ) and 4,4′-azo-1,2,4-triazole (4,4′-azo-1,2,4-triazole, ATRZ) as ligands, a variety of three-dimensional graphene-based energetic coordination polymers were synthesized by reaction with GO-Cu (II) complexes and additional metal ions. Two typical graphene-based high nitrogen compounds were synthesized, the molecular structure was shown in Figure 6. GO was reacted with Cu(NO_3_)_2_ in an aqueous solution to obtain graphene-Cu (II) complex, and if the reaction temperature is controlled above 60 °C, the yield will be higher.

## 3. Reduced Sensitivity of Nano-Graphene on Energetic Materials

According to the explosive hot spot theory, the process of explosive reaction causing an explosion is divided into three steps. Firstly, the hot spot is generated under the action of an external stimulus. Then the hot spot as the core causes the reaction to diffuse to the surrounding explosives, and gradually changes to low-speed combustion. After that, the low-speed combustion slowly grows to stable detonation and further produces an explosion. It can be seen that explosive explosion must meet two conditions, hot spot generation and propagation. As long as the two conditions of explosive explosion can be inhibited, that is, reduce the occurrence of a hot spot or cut off its transmission path. In this situation, explosives can be insensitive and prevent the further occurrence of explosions.

With the development of carbon nanomaterials, fullerenes, carbon nanotubes, nanocrystalline diamond and graphene have been used to reduce the sensitivity of explosives. Compared with fullerenes, carbon nanotubes and nano-diamonds, the application advantages of graphene oxide-based materials in the desensitization of energetic materials are more obvious. There are abundant oxygen-containing functional groups such as hydroxyl group, carboxyl group, epoxy group and carbonyl group on the surface and edge of graphene, which weakens the van der Waals force between graphene layers and makes it easy to slide between the layers, thus reducing the probability of hot spots. In addition, graphene has similar properties to polymers, gels and films, with high flexibility. Therefore, when the energetic material is stimulated by external stimuli, graphene can act as a buffer to prevent the formation and development of hot spots.

### 3.1. Reduced Sensitivity of Graphene-Based HMX Compound

**Figure 7:**
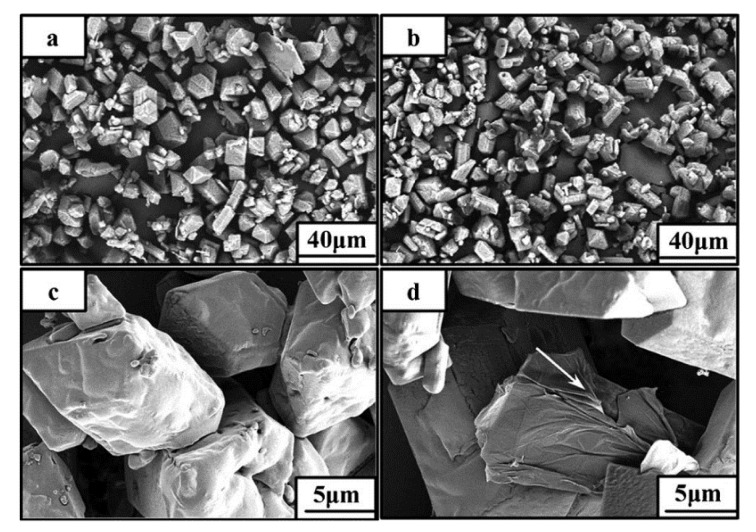
SEM images of HMX (**a**,**c**) and HMX/grapheme (**b**,**d**). Reprinted with permission from ref. [24]. Copyright 2013 John Wiley and Sons.

Li Rui et al. [24] prepared HMX/graphene energy-containing complex by solvent/anti-solvent method. Figure 7 shows SEM images of HMX and HMX/graphene composites, from which it can be seen that the surface of HMX/ graphene composites is smoother. After being combined with graphene, significant folds can be seen on the surface, indicating that graphene is stacked on HMX. It was found that the impact sensitivity of HMX decreased from 100% to 10% and the friction sensitivity decreased from 100% to 32% when 2% of the mass fraction of graphene was added. Graphene can significantly reduce the mechanical sensitivity of HMX compared to carbon nanotubes. Wang Jingyu et al. [27] also studied the effect of graphene on HMX, using viton and graphene as a coating agent and insensitive agent respectively, and using the water suspension method to coat HMX. The impact sensitivity, shock wave sensitivity and thermal decomposition performance of different coated samples were studied. The impact sensitivity of HMX, HMX/Viton, and HMX/Viton/graphene samples was 19.60 cm, 47.66 cm and 48.98 cm, respectively. The shock wave sensitivity of HMX/Viton, HMX/Viton/graphene samples were 9.25 mm, 7.49 mm, respectively. According to the above results, the impact sensitivity and shock wave sensitivity of HMX were significantly reduced after graphene coating, indicating that graphene can effectively reduce the sensitivity of HMX and improve its safety.

### 3.2. Reduced Sensitivity of Graphene-Based RDX (Cyclotrimethylenetrinitramine) Compound

**Figure 8:**
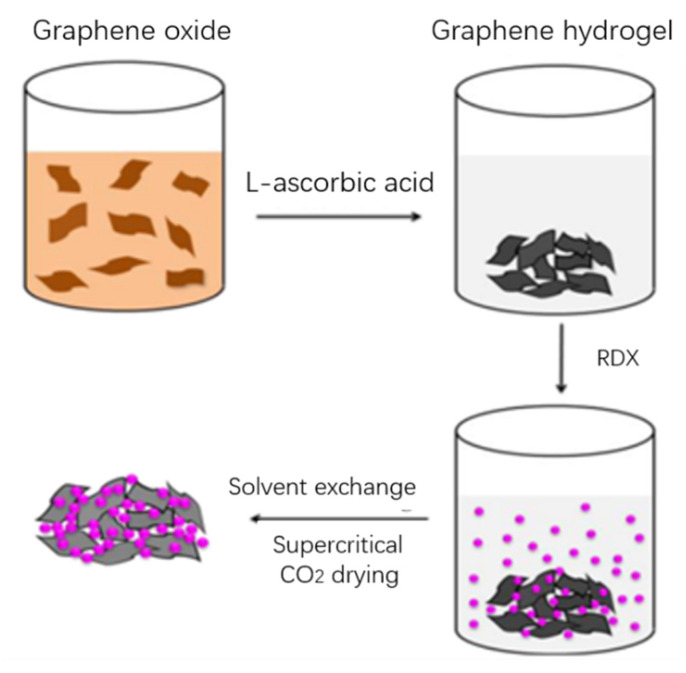
Schematic diagram of the preparation of GA/RDX nanocomposites [30]. Reprinted with permission from ref. [30]. Copyright 2016 Springer Nature.

Hu Fei et al. [28] selected RDX as the main explosive and graphene as the additive to study the effect of the proportion of additives on the properties of RDX. It can be seen from the experimental results that graphene can reduce the friction sensitivity of RDX within a certain range, and also reduce the explosion heat and explosion pressure of RDX (when the content is 7%, the explosion speed decreases 22.4%). This is because graphene consumes some of its total energy to burn itself during the explosion. Zorainy et al. [29] prepared the graphene-RDX composite using the solvent/anti-solvent method. Compared with three kinds of Polymer-bonded explosives based on RDX (PBXs), the impact sensitivity of the graphene-RDX composite containing 2 wt% graphenes was lower than that of the PBXs containing 9 wt% RDX. The inductance of graphene-RDX composite is similar to that of RDX-PMMA but higher than that of RDX-Viton and RDX-Fluorel. The results showed that graphene had a significant effect on RDX. The activation energy of the graphene-RDX complex is higher than that of RDX-Fluorel and RDX-Viton. The strong interaction between graphene and RDX makes the graphene-RDX complex have high thermal stability. Figure 8 is a schematic diagram of the preparation process of GA/RDX [30].

### 3.3. Reduced Sensitivity of Graphene-Based CL-20 Compound

**Figure 9:**
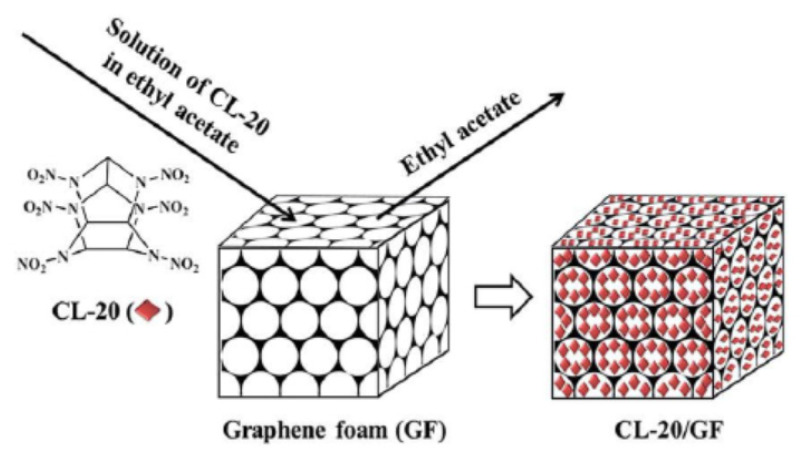
Schematic diagram of the production of CL-20/GF. Reprinted from ref. [31].

Baoyun Ye et al. [23] peeled graphite into graphene in the aqueous suspension of CL-20 and prepared CL-20/graphene-based composites by ball milling method. Compared to CL-20, CL-20/graphene-based composites have lower impact sensitivity, which is due to the excellent thermal conductivity and lubrication of graphene-based materials, which can effectively reduce the folding, dislocations and hot spots inside CL-20. Li Zhimin et al. [31] studied the effect of graphene foam on Cl-20 and prepared CL-20/graphene foam host-guest composite energetic materials by in situ crystallization method. The synthesis diagram is shown in Figure 9. Due to the induction of graphene foam, Cl-20 crystals are evenly distributed in their 3D skeleton. The addition of 2% graphene foam can reduce the thermal decomposition temperature and apparent activation energy of CL-20, improve the safety of CL-20, and maintain the excellent energy performance of CL-20. The detonation pressure and detonation velocity of the composite system are 44.1 GPa and 9687 m/s, respectively. Graphene-based materials can also reduce the sensitivity of CL-20. Graphene can effectively reduce the impact sensitivity of CL-20. The characteristic drop height (H_50_) of CL-20 is about 17.3 cm, while the characteristic drop height of composite materials containing 5% GO has increased to more than 150 cm [32]. Jin Zhenhua [33] used the recrystallization method to compound 2% of Gr, rGO and NGO with CL-20 respectively. After analysis, it was found that rGO had the most obvious desensitization effect on CL-20, and the critical drop height H_50_ was increased from 25.1 cm to 32.5 cm, the friction sensitivity is reduced from 100% to 72%. Song Xiaomin [34] proposed the desensitization mechanism of graphene oxide and polyethyleneimine on CL-20. She believes that the thermal conductivity and lubricity of graphene can slow down external mechanical or thermal stimulation. In addition, the amino groups carried by polyethyleneimine can form hydrogen bonds with nitro groups in CL-20 molecules to improve the charge characteristics of CL-20.

## 4. Thermal Decomposition and Combustion Behavior of Nano-Graphene-Based Materials

Thermal decomposition and combustion behavior are important characteristics of energetic materials when the materials are used in the propellant. The theoretical calculation, thermal decomposition and combustion behavior are reviewed as follows.

### 4.1. Molecular Dynamics Simulation of Nano-Graphene-Based Energetic Compounds

Since ReaxFF (a commonly used molecular dynamics simulation method) was used to simulate molecular dynamics (MD) [35,36,37,38], it has been widely used in the study of the thermal decomposition process of energetic materials because it can analyze the molecular changes in the thermal decomposition reaction of energetic materials from the microscopic perspective.

**Figure 10:**
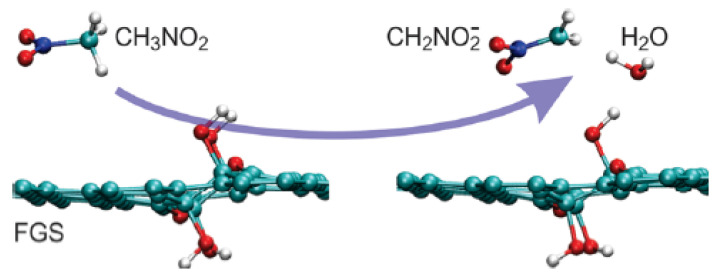
Initial decomposition of nitromethane on graphene sheets. Reprinted with permission from ref. [39]. Copyright 2012 American Chemical Society.

The molecular dynamics method was used to study the improvement of the combustion rate of nitromethane by functionalized graphene sheets [39]. The results showed that the vacancy defect of graphene sheets, namely the functionalized part of oxygen-containing groups, could promote the thermal decomposition of nitromethane and its derivatives, whose final products were H_2_O, CO_2_ and N_2_. The initial decomposition process is shown in Figure 10.

**Figure 11:**
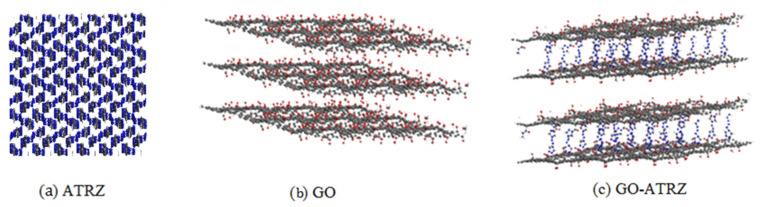
Molecule structure of 4,4′-azo 1,2,4-triazole (**a**), GO (**b**), and GO-4,4′-azo 1,2,4-triazole (**c**). Reprinted from ref. [40].

In order to evaluate the effect of GO on the thermal decomposition of 4,4′-azo-1,2, 4-triazole. Fu Xiaolong et al. [40] studied the thermal decomposition behavior of GO, 4,4′-azo-1,2,4-triazole (ATRZ) and GO-4,4′-azo-1,2,4-triazole complexes using molecular dynamics simulation of the ReaxFF reaction field, as shown in Figure 11. The results showed that the addition of GO increased the decomposition activation energy of 4,4′-azo-1,2,4-triazole. It can be inferred from the increase in activation energy that GO has a passivation effect on the decomposition of 4,4′-azo-1,2,4-triazole. In addition, the activation energy of the reaction to produce hydroxyl radicals in GO was 16.8 kJ·mol^−1^, indicating that the hydroxyl groups on the GO surface were easily detached.

**Figure 12:**
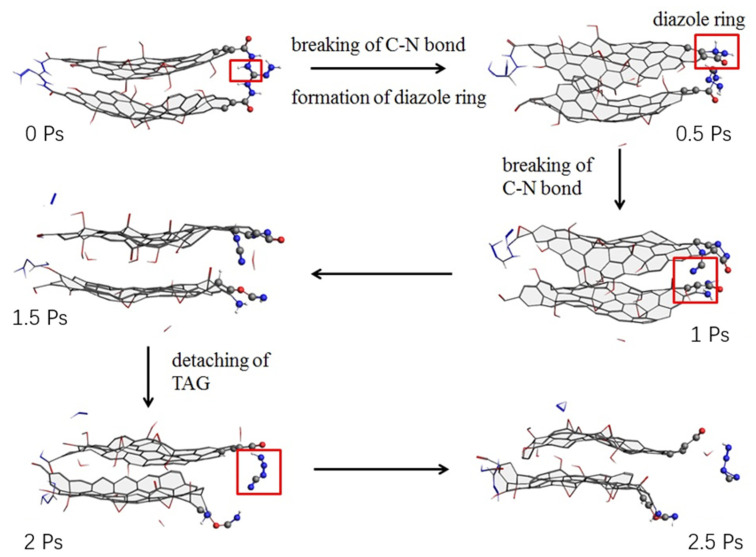
Molecular evolution of GO triaminoguanidine. Reprinted from ref. [40].

Zhang Chongmin [41] calculated the initial thermal decomposition mechanism and thermal decomposition products of triaminoguanidine (TAG) molecule and GO triaminoguanidine molecule by ReaxFF simulation. The results show that the decomposition products of GO are H_2_ and H_2_O at temperatures lower than 2500 K, and the C=O bond at the edge of GO breaks at 3000 K to form CO_2_ and CO. During the decomposition process, small sheets of GO tend to aggregate into large carbon clusters. The main decomposition products of triaminoguanidine are NH_3_, N_2_ and H_2_. The decomposition process of GO triaminoguanidine is similar to go, forming carbon clusters. The decomposition products are H_2_O, NH_3_, N_2_ and H_2_. During the decomposition process of triaminoguanidine, HN_2_, H_2_N and H radicals are generated. The initial decomposition activation energy of triaminoguanidine was 94.0 kJ∙mol^−1^, and that of triaminoguanidine in GO triaminoguanidine was 78.6 kJ∙mol^−1^. In other words, GO reduced the activation energy of triaminoguanidine by 15.4 kJ∙mol^−1^. By analyzing the initial decomposition process of triaminoguanidine and GO triaminoguanidine, it was found that the initial decomposition of triaminoguanidine was mainly due to intramolecular hydrogen transfer. The catalytic effect of GO on the initial decomposition of triaminoguanidine was mainly reflected in the carbon atoms at the edge of GO, which promoted the decomposition of triaminoguanidine molecules. Figure 12 shows the molecular evolution of Go-triaminoguanidine.

### 4.2. Thermal Decomposition and Combustion Behavior of Nano-Graphene-Based Energetic Compounds

Graphene has good combustion properties. At the same time, graphene can be evenly dispersed in water and organic solvents, which is conducive to full recombination with energetic materials. Therefore, graphene has a significant advantage in accelerating the reaction rate and energy release of energetic materials compared to other carbon materials. First of all, graphene has good electrical and thermal conductivity, which can effectively catalyze the thermal decomposition of energetic materials. Secondly, the energetic material forms nano-sized particles in the porous structure, which promotes the mass and heat transfer process of the material and greatly improves the energy release rate of the system. Thirdly, graphene, as a carbon material, is able to react with oxidants to contribute more heat without creating residual particles.

Wang [21] studied the effect of graphene on the thermal decomposition of ammonium perchlorate. Compared with ammonium perchlorate, the heat decomposition temperature of ammonium perchlorate was 66.9 °C in advance in the case of ammonium perchlorate. In addition, the total apparent decomposition of ammonium perchlorate was 621 J/g, and the total apparent decomposition of ammonium perchlorate and graphene was 1786 J/g. graphene significantly improved the apparent decomposition of ammonium perchlorate. The results show that graphene can significantly promote the thermal decomposition of AP. In addition, there is a large number of pro-nuclear oxygen groups in the graphene layer and edge of the oxide graphene layer, which is easy to make with the ability to modify and the perfect treatment of the physical properties of graphene and the material. Zhang et al. [42] used a mixture of nitric acid and sulfuric acid to nitrate graphene oxide to prepare nitrated graphene and introduced ammonium perchlorate to study the catalytic effect of ammonium perchlorate. The thermal decomposition temperature of ammonium perchlorate was reduced by 106 °C, and the apparent decomposition of heat from 875 J/g increased to 3236 J/g. The catalytic effect of ammonium oxide on ammonium perchlorate, which can effectively reduce the heat decomposition temperature of ammonium perchlorate, and improve the apparent decomposition of ammonium perchlorate. In addition, the unique band structure can accelerate the electron transfer of the rapid rate control step of high chlorate and high-temperature decomposition process (the rapid control step of the low-temperature decomposition is transferred from ClO_4_^−^ to NH_4_^+^, and the speed control step of the high-temperature decomposition is transferred from O_2_ to the superoxide O_2_^−^), which can improve the rate of reaction rate [43].

LAN Yuanfei et al. [30] prepared graphene aerogel by sol–gel method and supercritical CO_2_ technology and then immersed RDX in graphene aerogel solution to prepare graphene aerogel/RDX nano-energy-containing composites. Due to the unique nano-porous structure of graphene aerogel, the growth of RDX is limited, which makes the nanoscale RDX evenly dispersed into the graphene aerogel. Graphene aerogels can significantly accelerate the decomposition of RDX, reduce the thermal decomposition temperature and accelerate the decomposition rate, which contributes to the full reaction of RDX.

**Figure 13:**
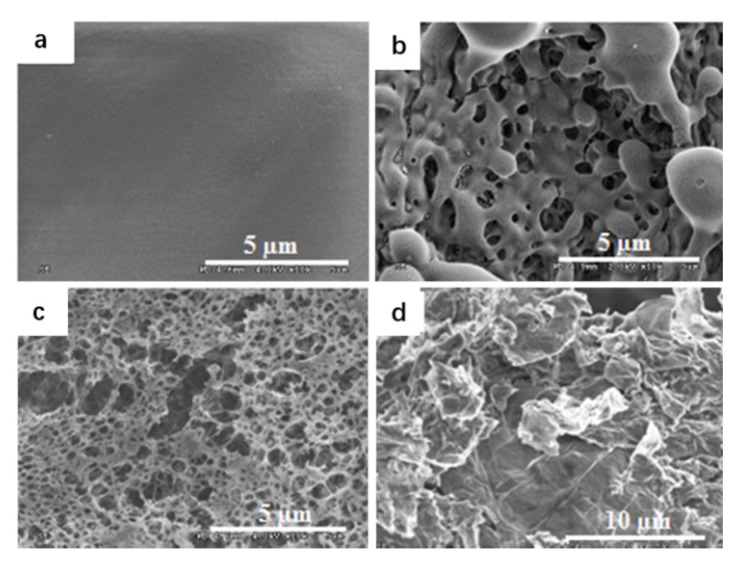
SEM images of pure and GO doped NC films. SEM image of (**a**) pure NC film, (**b**) 0.5% and (**c**) 2% GO doped NC film; (**d**) as-produced GO. Reprinted with permission from ref. [44]. Copyright 2013 AIP Publishing.

Zhang et al. [44] used the solvent/anti-solvent method to compound GO and nitrocellulose, transforming NC (nitrocellulose) from fibrous structure to three-dimensional network structure. Nitrocellulose films doped with GO were ignited by a 20 ns laser at low temperatures. It was found that GO could significantly improve the ignition and combustion characteristics of micron-sized Nitrocellulose films. The researchers also investigated micron-sized nitrocellulose films doped with different concentrations of GO (0.1%, 0.5%, 1% and 2%). Figure 13 represents the different morphologies of the Pure NC and GO-doped NC films as observed by SEM. The pure nitrocellulose film is continuous, smooth and homogeneous. When GO was added to nitrocellulose film, the surface morphology changed dramatically and the surface became very rough.

Zhao Fengqi et al. [45] prepared reduced graphene/Fe_2_O_3_ composites by hydrothermal and solvothermal methods. Reduced graphene/Fe_2_O_3_ had a significant promoting effect on the thermal decomposition of 5,5′-ditetrazole-1,1′-dioxy-dihydroxylammonium (TKX-50), reducing the low-temperature decomposition peak temperature of TKX-50 and increasing the apparent decomposition heat. This is because nano Fe_2_O_3_ particles are loaded on graphene, and the dispersibility is significantly improved, which can provide more active sites during the catalytic process and promote the decomposition of TKX-50.

Yuan Shen et al. [46] studied the reaction mechanism of NC catalyzed by nitrographene. He found that an important factor limiting the reaction rate of NC thermal decomposition was the low content of NO_2_ generated by NC self-decomposition, and the addition of nitrographene could provide NO_2_ for the reaction, thus increasing the reaction rate and the apparent heat of decomposition greatly.

**Figure 14:**
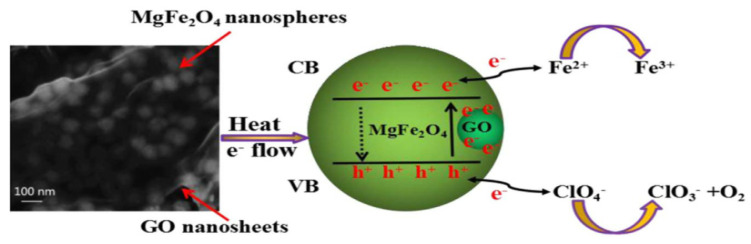
Schematic diagram of thermal catalytic activity mechanism of GO coated MgFe_2_O_4_ composite. Reprinted with permission from ref. [44]. Copyright 2013 AIP Publishing.

Zu et al. [47] a composite catalyst containing GO and found that the catalyst could effectively catalyze the thermal decomposition reaction of AP. By analyzing its mechanism, they found that the role of GO was to reduce the decomposition temperature of AP by increasing the transfer rate of electrons in AP from ClO_4_^−^ to NH_4_^+^. Figure 14 shows the catalytic mechanism of GO-coated MgFe_2_O_4_ composite.

In order to investigate the effect of graphene on the reaction mechanism of energetic materials, Liu Yingzhe et al. [48] established a graphene/nitromethane model and explored the effect of graphene on energetic materials using combinational quantum chemistry ONIOM (our Own N-layer Integrated molecular Orbital and molecular Mechanics) method. The results show that graphene can induce the reaction of nitromethane, and nitromethane has hydrogen rearrangement on the surface of graphene, which increases the interaction between them.

## 5. Applications of Nano-Graphene-Based Materials in Propellants and Explosives

The application of graphene-based compounds in solid propellants is one of the most concerning issues for researchers. The effect of graphene-based compounds on propellant combustion is the key factor for their application [49,50]. At present, countries including the United States and India have carried out research on the application of graphene-based compounds in propellants.

Nasir K. Memon [51] in Purdue University was prepared an energetic composite material based on graphene oxide and ammonium perchlorate. The resulting composite material is found to enhance the thermal decomposition of ammonium perchlorate over just physically mixing. Furthermore, using this material, bimodal AP/hydroxyl-terminated polybutadiene solid propellants were prepared. The combustion behavior of the propellants was investigated, and the graphene-oxide-based solid propellant resulted in a 15% increase in the burning rate at a pressure of 80 atm over the baseline material.

**Figure 15:**
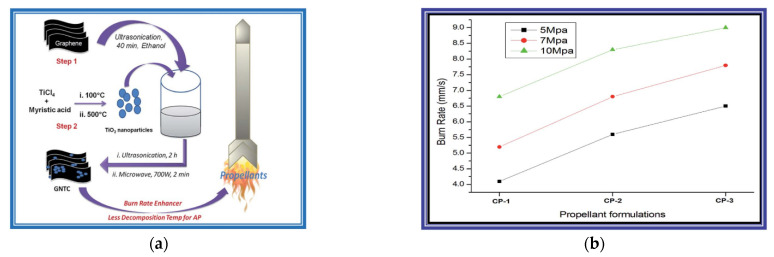
(**a**) Schematic representation of the synthesis and application of the graphene–titanium dioxide nanocomposite (GTNC) (**b**) Variation of the burn rate with pressure for different propellant compositions. Reprinted from ref. [52].

Abhijit Dey in Deemed University [52] synthesis graphene–titanium dioxide nanocomposite (GTNC). The GTNC was identified as an effective burn rate enhancer for an AP-based composite propellant for solid rocket propellants. The results show that the burning rate of the propellant increases by 24% for the TiO_2_ nanoparticle-based composition compared to the base composition, whereas a significant increase of 50% is achieved in the presence of the GTNC. Hence, the performance is improved significantly for the solid rocket propellant. The specific experimental results of the propellant are shown in Figure 15.

**Figure 16:**
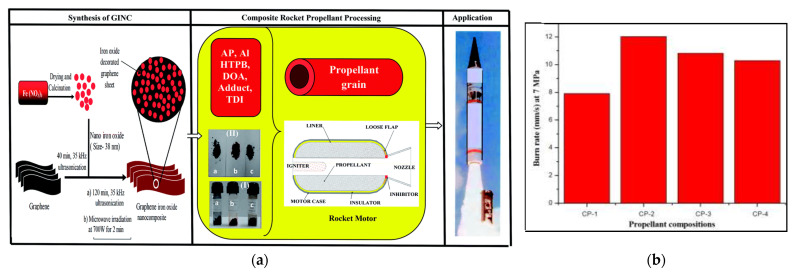
(**a**) Synthesis, processing and application of GINC as burn rate modifier in missile system; (**b**) burn rate of propellant. Reprinted from ref. [53].

Abhijit Dey in Deemed University [53] also synthesized nano size iron oxide (Fe_2_O_3_) decorated graphene (GINC) hybrid materials, and further extend this application as a burn rate enhancer in the composite propellant. Figure 16a shows the synthesis, processing and application of GINC as a missile system combustion rate regulator. The results show that the burn rate of propellant increases from micron size Fe_2_O_3_ (30% increases) to nano Fe_2_O_3_ (37% increase). In the presence of GINC, a significant increase (52%) in burn rate is achieved. In GINC, 20 effective iron content is about 50% as compared to nano and micron size Fe_2_O_3_. Hence GINC was found to be an excellent burn rate modifier for advanced AP-based propellant systems. (CP-1: Binder (15%) + Al(17%) + AP(68%) + Burn rate enhancer(Fe_2_O_3_)-Nil, CP-2: Binder (15%) + Al(17%) + AP(68%) + Burn rate enhancer(GINC)-1 wt.% over the batch, CP-3: Binder (15%) + Al(17%) + AP(68%) + Burn rate enhancer(nano Fe_2_O_3_)-1 wt.% over the batch, CP-4: Binder (15%) + Al(17%) + AP(68%) + Burn rate enhancer(Micron size Fe_2_O_3_)-1 wt.% over the batch). The burning rates of the four propellants are shown in Figure 16b.

**Figure 17:**
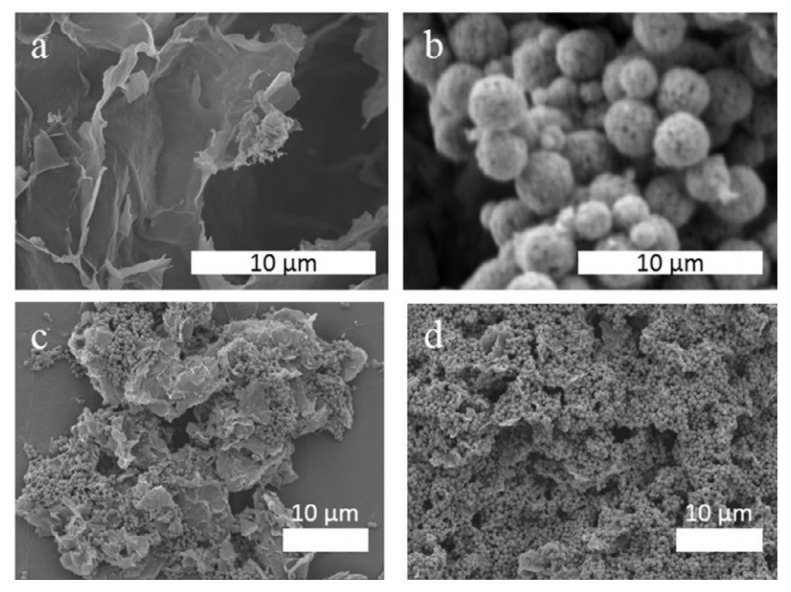
SEM images of (**a**) blank graphene, (**b**) iron oxide, (**c**) NH_2_^−^functionalized decorated graphene, and (**d**) SO_3_H^−^functionalized decorated graphene. Reprinted with permission from ref. [54]. Copyright 2017 Elsevier.

**Figure 18:**
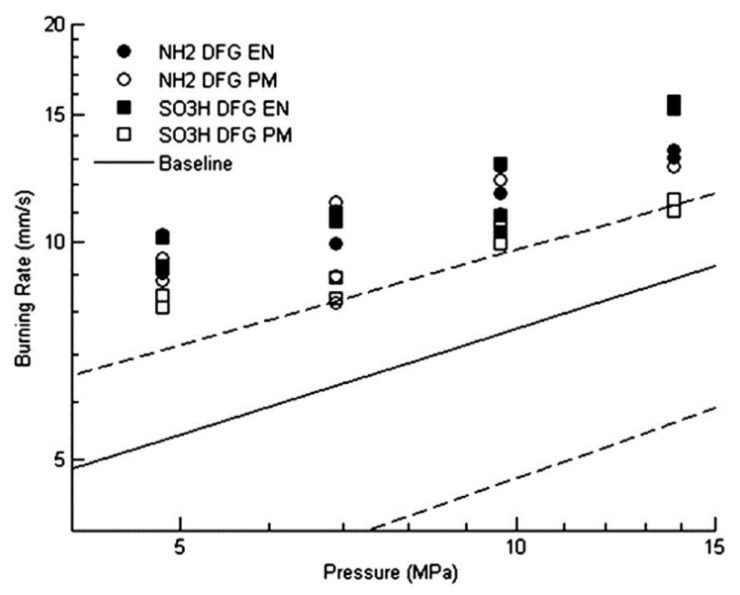
Burning rates of propellants with decorated graphene. Open symbols represent physical mixes and closed symbols encapsulated catalysts. Solid line is the baseline propellant burning rate, and dashed lines are the 95% confidence interval for the baseline propellant burning rate. Reprinted with permission from ref. [54]. Copyright 2017 Elsevier.

S. Isert [54] in Purdue University compared propellants with decorated graphene and propellants with undecorated graphene catalysts. SEM images of graphene and functionalized graphene are shown in Figure 17. Graphene decorated with iron oxide did cause a change in the burning rate. Both the encapsulated and physically mixed cases resulted in a large (>25%) increase in burning rate over the baseline propellant, and in some cases, the increase was almost 100%. Figure 18 shows that when using decorative graphene, there is a difference in the burning rate of the physically mixed propellant and the encapsulated particle propellant.

## 6. Characterization of Nano-Graphene-Based Energetic Materials

In this paper, we also summarized some commonly used characterization methods of graphene-based energetic compounds in Table 1.

## 7. Conclusions

Nano-graphene-based energetic compounds are a kind of multifunctional component of propellants and explosives with excellent comprehensive properties. At present, researchers have carried out a large number of studies on nano-graphene-based energetic compounds, but the research on nano-graphene-based energetic compounds still has the following problems:

(1) The research on the reaction mechanism and structure design of the preparation of nano-graphene-based energetic compounds is still unclear.
(2) The sensitization mechanism of nano-graphene on energetic compounds is still unclear.
(3) The engineering application of nano-graphene-based energetic compounds still needs long-term exploration.

In conclusion, the molecular structure of nano-graphene-based energetic compounds is complex and elucidating the relationship between their structure and thermal stability will help to further understand the main effects and mechanisms of graphene-based compounds in propellants. Therefore, the research on the hot point inhibition principle, thermal decomposition characteristics and thermal decomposition mechanism of nano-graphene-based energetic compounds are of great practical significance in guiding the design, preparation and application evaluation of new graphene functional materials.

## Figures and Tables

**Table 1:** Characterization of graphene-based energetic compounds.

Methods	Content	Examples	Analysis Results	Ref.
SEM	Morphology, size	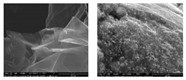	Fe_2_O_3_ is adsorbed on rGO lamellar.	Reprinted from ref. [45]
TEM	Morphology, size	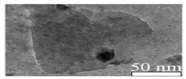	Lamellar stripping of GO.	Reprinted from ref. [55]
EDS	Element distribution	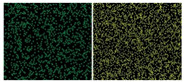	Large, uniform amounts of carbon (left) and oxygen (right)	Reprinted from ref. [56]
AFM	Thickness	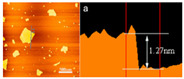	The apparent thickness of GO is about 1.27 nm.	Reprinted with permission from ref. [42]. Copyright 2014 Elsevier
FTIR	Thermal decomposition product	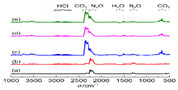	The thermal decomposition products are HCl, N_2_O and H_2_O.	Reprinted from ref. [57]
Raman	The chemical structure	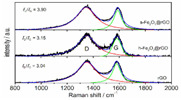	Fe2O3 increases defects in graphene.	Reprinted from ref. [45]
XRD	The crystal structure	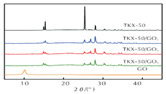	GO content has little effect on tKX-50 crystal shape.	Reprinted from ref. [58]
DSC	Thermal performance	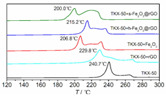	The graphene additive reduces the decomposition temperature of TKX-50.	Reprinted from ref. [45]
TG-FTIR	Thermal decomposition product	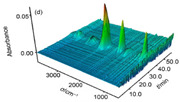	The thermal decomposition products are HCl, N_2_O, H_2_O and CO_2_.	Reprinted from ref. [57]

## Data Availability

Data are contained within the article.
